# Efficacy of botulinum toxin for poststroke lower limb: a systematic review and meta-analysis

**DOI:** 10.1177/02692155261417499

**Published:** 2026-02-13

**Authors:** Carla Sílvia Fernandes, Andreia Maria Novo Lima, Maria Teresa Moreira, Salome Sobral Sousa, Maria Salomé Ferreira

**Affiliations:** 1 112031 School of Health, Polytechnic Institute of Viana do Castelo, Portugal; 2 Research Center RISEHealth, Portugal; 3 Association ADITGames, Portugal; 4 Health Sciences School - Fernando Pessoa, Porto, Portugal 70990; 5 Research Center for Health Technologies and Services (RISE@Health), Porto, Portugal; 6 112085 Nurse Department, University Hospital Center of Porto EPE (CHUP), Porto, Portugal; 7 112031 School of Health, Polytechnic Institute of Viana do Castelo, Viana do Castelo, Portugal; 8 The Health Sciences Research Unit: Nursing (UICISA:E), Coimbra, Portugal

**Keywords:** Stroke, botulinum toxin, spasticity, lower limb, rehabilitation

## Abstract

**Objective:**

This review aims to conduct a systematic review and meta-analysis on the effectiveness of botulinum toxin in the treatment of spasticity and the improvement of lower limb function in adult stroke survivors, based on randomized clinical trials.

**Data sources:**

Searches were conducted across multiple databases, including Medline, Scopus, CINAHL, SPORTDiscus, Psychology and Behavioral Sciences Collection, and Cochrane, from inception until September 2025.

**Review Method:**

This systematic review and meta-analysis was reported in accordance with the Preferred Reporting Items for Systematic Reviews and Meta-Analyses recommendations. The meta-analysis was performed using Review Manager 5 software, with mean differences pooled using a random-effects model.

**Results:**

The analysis included 11 studies, with a total of 1740 adult participants. The results confirm that botulinum toxin is effective in reducing spasticity; however, the benefits on other outcomes, namely gait and balance, were limited and inconsistent. Considerable heterogeneity was also observed in intervention protocols, participant characteristics, dosing regimens, and the selection of target muscle groups.

**Conclusion:**

It is recommended that future studies prioritize the inclusion of subgroups and medium- and long-term follow-up. Only in this way will it be possible to clarify the true efficacy of botulinum toxin in different patient profiles and to contribute to the optimization of lower limb rehabilitation strategies after stroke.

## Introduction

Spasticity is a common complication following stroke, often leading to significant impairments and functional limitations.^1,2^ Stroke remains the second leading cause of mortality and the third leading cause of combined death and disability worldwide.^3–5^ Between 1990 and 2021, the global burden of stroke, measured by the number of cases, deaths, and individuals living with disability, increased substantially.^2^ Poststroke consequences can include difficulties with activities of daily living, mobility impairments, balance disorders, mood and cognitive changes, pain, incontinence, and spasticity.^6,7^

The global prevalence of poststroke spasticity is estimated at 25.3%.^2^ Spasticity is defined as a velocity-dependent increase in muscle tone accompanied by exaggerated stretch reflexes, posing a substantial barrier to functional recovery.^8^ It may present in various patterns such as focal, multifocal, segmental, hemispastic, paraspastic, or generalized.^1^ A comprehensive and systematic assessment is essential, as spasticity often coexists with other motor deficits such as weakness, poor motor control, and abnormal synergies.^1,9,10^

There is increasing recognition of the importance of controlling spasticity, not only to facilitate mobility but also to prevent secondary complications.^1^ However, despite advances in treatment, the overall effectiveness of available therapies remains controversial.^1,8^ While reducing muscle tone is often the immediate therapeutic goal, the broader objective should be the enhancement of functional performance.^9–12^ Approximately one-third of stroke survivors develop lower limb spasticity, most commonly affecting the ankle plantar flexors, hip adductors, knee extensors, followed by knee flexors and hip internal rotators, substantially impacting daily activities and quality of life.^13^ Importantly, the mere presence of spasticity does not automatically justify treatment.^1,9^ Therapeutic intervention is warranted when spasticity significantly limits range of motion or meaningfully impairs function, even when muscle tone elevation is relatively mild.^1^ Spasticity management typically follows a phased approach, starting with postural strategies and orthotic devices, progressing to pharmacological interventions, and, when indicated, targeted approaches such as botulinum toxin (BTX) injections or other advanced treatments.^1,11^ Among therapeutic options, BTX type A is currently considered the gold standard for focal spasticity.^9,12^ However, despite its wide acceptance and favorable safety profile, concerns remain regarding its cost-effectiveness and its actual impact on functional recovery in lower limb spasticity.^1,9,11^

Given the functional burden associated with lower limb spasticity poststroke, a critical evaluation of BTX as a therapeutic tool is warranted. Although it is commonly recommended and widely used, there is ongoing uncertainty about its effectiveness in improving functional outcomes in stroke survivors,^1,8,13^ particularly when compared with conventional or adjunctive interventions. Previous reviews often included heterogeneous samples, combining upper and lower limb spasticity,^14,15^ mixing stroke with other neurological conditions,^14,16^ or incorporating combined treatments such as BTX and electrical stimulation.^17^ In this study, trials combining BTX with other rehabilitation technologies were excluded in order to isolate the specific effect of the pharmacological agent and to reduce methodological heterogeneity, thereby enabling a more robust interpretation of the findings.

In this context, conducting a systematic review and meta-analysis focused exclusively on randomized controlled trials (RCTs) is essential to clarify the isolated therapeutic effects of BTX. This will support more robust clinical recommendations regarding its use in the treatment of lower limb spasticity in adult stroke survivors.

## Methods

### Study design

The present systematic review and meta-analysis was developed in accordance with the methodological recommendations of the Joanna Briggs Institute.^18^ The study design followed the criteria of the PRISMA (Preferred Reporting Items for Systematic Reviews and Meta-Analyses) checklist,^19^ using the PRISMA flow diagram to illustrate the study selection process,^20^ thereby ensuring transparency and rigor in the processes of study selection, appraisal, and data synthesis. The research protocol is registered on the OSF platform (https://doi.org/10.17605/OSF.IO/TN5KH).

### Search strategy

The search was conducted across several electronic databases, including Medline, CINAHL, Scopus, Psychology and Behavioral Sciences Collection, Cochrane, and SPORTDiscus (Supplementary Material). To ensure a more comprehensive selection of relevant studies, the reference lists of the selected articles were also analyzed using a strategy known as “backward citation,” meaning that the reference lists of all included studies were manually screened to identify any additional eligible publications.

### Inclusion and exclusion criteria

Studies involving adult participants (aged 18 years or older) diagnosed with stroke were considered eligible if the primary intervention consisted of the isolated administration of BTX, regardless of the subtype or formulation used, with application directed exclusively to the lower limb(s). Randomized clinical trials, controlled trials, or comparative clinical studies published in full were included.

Studies were excluded if they involved pediatric participants (under 18 years of age) or participants with other conditions resulting in spasticity, such as traumatic brain injury or other etiologies. Studies were also excluded if the intervention did not target the lower limb or if BTX was administered simultaneously with other resources, such as robotics, virtual reality, shockwave therapy, or any other treatments performed concurrently with toxin administration. Studies in which it was not possible to isolate the effect of BTX due to the concomitant use of other therapeutic interventions (except for conventional physiotherapy equally applied in all groups) were also excluded. Publications in languages other than those previously established and studies that did not present quantitative or qualitative data regarding outcomes of interest for the lower limb were likewise excluded. Articles published in Spanish, English, or Portuguese from the inception of these databases until September 2025 were included.

### Review process

The search results were uploaded to the Rayyan QCRI^®^ platform (https://rayyan.qcri.org/, Qatar). During the initial screening, three researchers independently reviewed the titles, keywords, and abstracts to identify potentially eligible studies. Whenever doubts or disagreements arose during the selection process, these were resolved by consensus, with the involvement of two additional authors to ensure a joint decision on the inclusion of publications. Subsequently, the articles deemed relevant were assessed in full to verify compliance with all inclusion criteria. At every stage of the process, any discrepancies were discussed until consensus was reached among the authors, ensuring eligibility and rigor in the selection of studies.

### Data extraction

**Figure 1.**
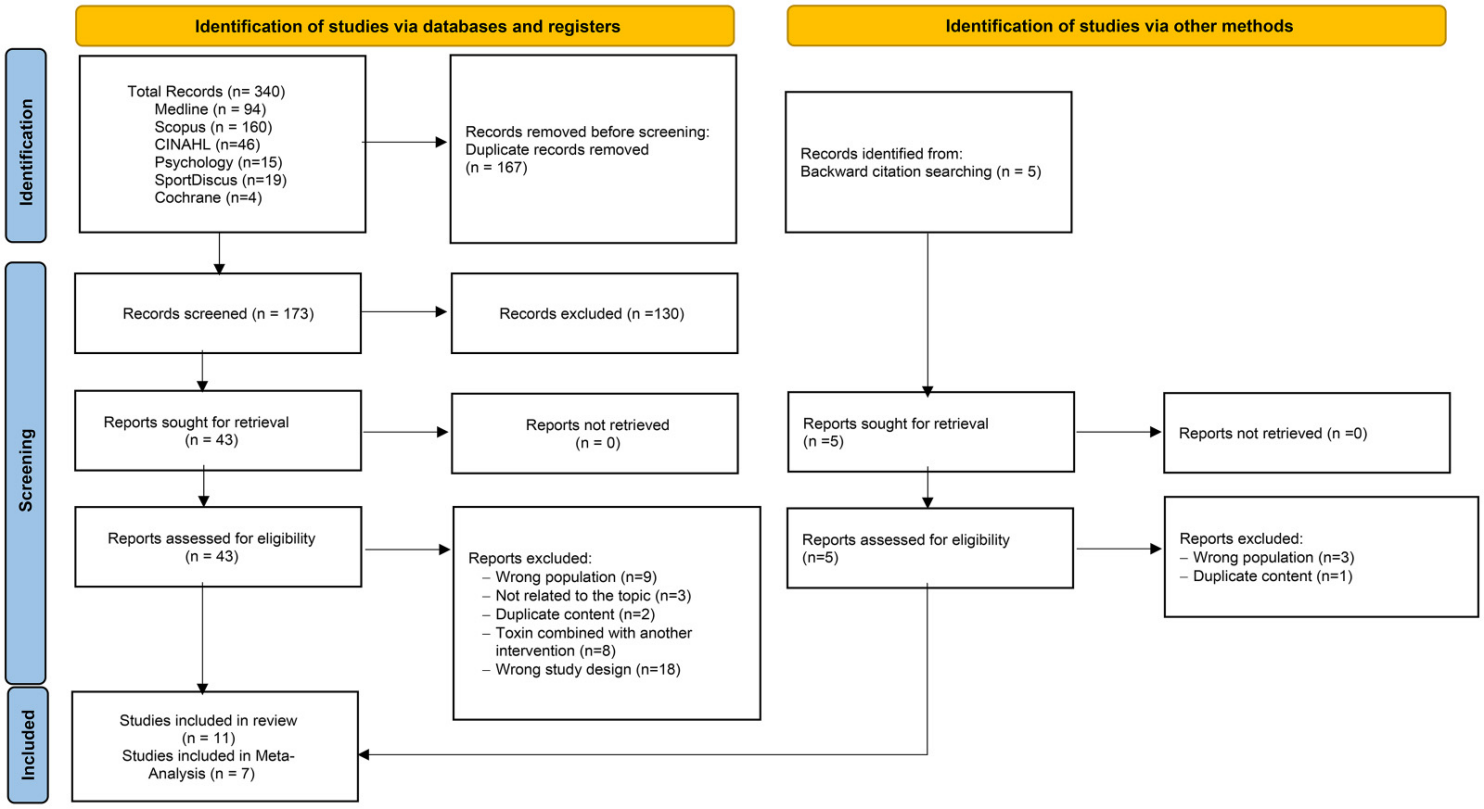
Preferred Reporting Items for Systematic Reviews and Meta-Analyses (#PRISMA) diagram flow 2020.

Figure 1 illustrates the process of article identification and inclusion, following the PRISMA^®^ guidelines. Data extraction was performed independently by two researchers, who collected essential information from each study. Whenever uncertainties arose during data extraction, these were discussed with the other researchers to ensure the accuracy of the information. In cases where articles had gaps or unclear data, the study authors were contacted to obtain clarification or additional information.

### Quality appraisal and risk of bias

The methodological quality of the included studies was assessed based on the procedures and criteria defined in the Cochrane Collaboration Handbook for Systematic Reviews of Interventions.^21^ This assessment covered six key domains: random sequence generation, allocation concealment, blinding of participants and intervention staff, blinding of outcome assessment, completeness of outcome data, and selective outcome reporting or other potential sources of bias. Three researchers conducted this analysis independently. When disagreements in study ratings arose, the data were discussed with another researcher.

### Statistical analysis

Initially, a data extraction table (Table 1) was developed to organize relevant information from the included studies. In a second phase, quantitative outcomes with methodological homogeneity and available data were grouped for meta-analysis. The remaining data are presented descriptively in Table 1.

**Table 1.** Data extraction.

Author (year) country	Study design	Participants							Intervention			Control group	Assessment tools
		*N*	Mean age (±SD)	Sex	Stroke phase	Stroke type	Affected side	Degree of spasticity	Type of toxin	Dose	Muscle group(s)		
Kaji et al. (2010) ^22^ Japan	Multicenter, randomized, double-blind, placebo-controlled trial	120 (Toxin: 58; Placebo: 62)	62.4 ± 8.7 (Toxin); 62.5 ± 9.3 (Placebo)	8F/50 M (Toxin); 16F/46 M (Placebo)	Chronic (≥6 months; mean 80.8 ± 72.8 months for Toxin; 72.0 ± 60.3 months for placebo)	Not specified	Not specified	MAS ∼3.8 (Toxin), 3.2 (Placebo)	OnabotulinumtoxinA (BOTOX^®^, Allergan)	300 U total 75 U each muscle, single session	Medial gastrocnemius, lateral gastrocnemius, soleus, tibialis posterior;	Placebo (saline, identical injection)	MAS, PRS, GS, CGI
Tok et al. (2012) ^23^ Turkey	Randomized, double-blind, placebo-controlled trial	*N* = 25 (toxin A: 15; Placebo: 10)	53.9 ± 14.7 (Toxin), 59.0 ± 8.1 (Placebo)	Toxin 7F/8 M, Placebo 4F/6M	Chronic ≥6 months; duration Toxin 14.6 ± 7.2 Placebo 13.4 ± 7.0	Ischemic 80%, Hemorrhagic 20%	Toxin 9R/6L, Placebo 5R/5L	(rectus femoris) 2.46 ± 0.63 (Toxin), 2.40 ± 0.51 (Placebo)	Onabotulinum toxin A (BOTOX^®^, Allergan),	mean dose 113.3 ± 12.9 U (range 100–125 U), single injection	Rectus femoris	Placebo (saline, identical injection)	MAS, 10MWT, 6MWT, 3D Gait, VO₂
Pittock et al. (2003) ^24^ Multicenter	Randomized, double-blind, placebo-controlled trial	*N* = 234 Placebo: 55 Dysport 500U: 59 Dysport 1000U: 60 Dysport 1500U: 60	Placebo: 55.9 (±11.4) 500U: 56.4 (±12.8) 1000U: 54.8 (±13.6) 1500U: 54.7 (±10.2)	Placebo: 37/18 500U: 36/23 1000U: 39/21 1500U: 35/25	Chronic (≥3 months poststroke)	Intracerebral hemorrhage, subarachnoid hemorrhage, cerebral thrombosis, cerebral embolism	Both sides included	MAS (mean ∼3.0, some up to 5)	BoNT-A (Dysport^®^)	500, 1000, 1500 Single injection per group	Gastrocnemius, soleus	Placebo (saline, identical injection)	MAS, 2MWT, RMA
Dunne et al. (2012) ^25^ Australia	Randomized, double-blind, placebo-controlled trial	*N* = 83 OnabotulinumtoxinA: 54 Placebo: 29	OnabotulinumtoxinA 57.9 ± 13.8 Placebo 59.5 ± 10.6	OnabotulinumtoxinA: 76% M Placebo: 76% M	Chronic Median 2.1 years (mean 3.4 ± 3.8 years), 22.4% < 6 months	74% ischemic, 26% hemorrhagic (overall)	Right: 60%, Left: 40%	Moderate-severe (Ashworth ≥2)	OnabotulinumtoxinA (Botox)	200 U or 300 U (pooled in analysis); max 300 U	Tibialis posterior, soleus, medial gastrocnemius, flexor digitorum longus	Placebo (saline, identical injection)	MAS, SFS, PRS, VAS
Esquenazi et al. (2018) ^26^ Multicenter	Multicenter, randomized, double-blind, placebo-controlled trial	*N* = 468 (OnabotulinumtoxinA: 233; Placebo: 235)	56.0 (±12.6) (ona), 57.0 (±11.9) (placebo)	148/85 (ona), 155/80 (placebo)	Chronic ≥6 months poststroke	Not specified	Both	MAS ≥3 (92% baseline MAS = 3)	OnabotulinumtoxinA (BOTOX^®^)	300–400 (mean 347.5) Single injection	Medial gastrocnemius, lateral gastrocnemius, soleus, tibialis posterior	Placebo (saline, identical injection)	MAS, CGI, GAS, 10MWT
Masakado et al. (2016) ^27^ Japan	Randomized, double-blind, placebo-controlled trial	*N* = 208 IncobotulinumtoxinA: 104 Placebo: 104	IncobotulinumtoxinA: 59.5 (11.2) Placebo: 58.8 (11.0)	IncobotulinumtoxinA: 74/30 Placebo: 84/20	Chronic (>6 months)	Ischemic: ∼30% Hemorrhagic: ∼70%	Unilateral (hemiparesis)	MAS-PF = 3 (severe)	IncobotulinumtoxinA (Xeomin^®^)	400 (fixed)	- Medial gastrocnemius - Lateral gastrocnemius - Soleus - Tibialis posterior - Flexor digitorum longus - Flexor hallucis longus	Placebo (saline, identical injection)	MAS, MAS-PF, 10MWT, PRS, CGI, VAS, NRS
Tao et al. (2015) ^28^ China	Randomized, double-blind, placebo-controlled trial	*N* = 23 (Toxin: 11; Placebo: 13)	Treatment: 55 ± 12 Control: 58 ± 14	Treatment: 7/4 Control: 8/4	Subacute (within 6 weeks)	Ischemic/hemorrhagic (Treatment: 6/5; Control: 7/5)	Not specified	MAS 1–1 + or ankle clonus (+)	Botulinum toxin A (Allergan, Botox)	200 U	Triceps surae (150 U), posterior tibial (50 U)	Placebo (saline, identical injection)	MAS, FMA, sEMG, MBI
Yu et al. (2023) ^29^ China	Randomized, double-blind, placebo-controlled trial	*N* = 46 (Toxin: 23; Placebo: 23)	Control: 54.4 ± 9.8 Experimental: 56.5 ± 6.8	Control: 8 F / 14 M Experimental: 5 F / 16 M	Subacute (≤6 months poststroke)	Cerebral infarct (32), cerebral hemorrhage (14)	Control: 12 L / 10 R Experimental: 13 L	Slight spasticity at rest: MAS 1–1 + or ankle clonus (+)	∼150–300 IU/muscle (see below)	200–300 U total	Quadriceps femoris, gastrocnemius, tibialis posterior, flexor hallucis longus, flexor digitorum longus, flexor digitorum brevis, flexor hallucis brevis	Routine rehabilitation	MAS, L-FMA, 10MWT, TUGT
Tenniglo et al. (2023) [30 Netherlands	Randomized, double-blind, placebo-controlled trial	*N* = 25 (crossover: BoNT-A and placebo)	57.4 (12.7)	19 M / 6 F	Chronic (≥6 months poststroke)	17 ischemic / 8 hemorrhagic	15 left / 10 right	Moderate to severe	OnabotulinumtoxinA (Botox, Allergan)	200 U (6 × 33U into rectus femoris)	Rectus femoris	Placebo (saline, identical injection)	3D Gait, 6MWT, 10MWT, TUG, MI, RMI, MRC, VAS, SIS
Wein et al. (2018) ^31^ Multicente	Randomized, double-blind, placebo-controlled trial	*N* = 468 (233 intervention / 235 placebo)	Intervention: 56.0 (12.6) Placebo: 57.0 (11.9)	Intervention: 148 M / 85 F Placebo: 155 M / 80 F	Chronic (≥3 months after stroke) Mean time since stroke: 67.1 mo (INT), 61.6 mo (PLA)	Not specified	Right only, left only, right arm/leg, left arm/leg	MAS ≥3	OnabotulinumtoxinA (Botox^®^)	300 U	gastrocnemius medial/lateral, soleus, tibialis posterior) + optional muscles, flexors/extensors, rectus femoris)	Placebo (saline, identical injection)	MAS, CGI, GAS, VAS, MTS
Kerzoncuf et al. (2020) ^32^ Farnce	Randomized, double-blind, placebo-controlled trial	*N* = 40 (19 BoNT-A / 21 placebo, analyzed)	BoNT-A: 53.4 ± 14.8 Placebo: 50.7 ± 12.9	Not specified	Chronic (≥12 months poststroke, mean ∼4–6 yrs)	Ischemic & hemorrhagic (proportion not specified)	Both (left/right, similar)	MAS ≥2	OnabotulinumtoxinA (Botox^®^)	Mean 227 U (range 50–300 U)	Soleus (88%), gastrocnemius (73%), tibialis posterior, flexor digitorum longus, flexor/extensor hallucis	Placebo (saline, identical injection)	MAS, ROM, FAC, FIM

3D Gait: Three-Dimensional Gait Analysis; 6MWT: 6-Minute Walk Test; 10MWT: 10-Meter Walk Test; 2MWT: 2-Minute Walk Test; CGI: Clinical Global Impression; FAC: Functional Ambulation Category; FIM: Functional Independence Measure; FMA: Fugl-Meyer Assessment; GAS: Goal Attainment Scale; GS: Gait Speed; L-FMA: Lower-limb Fugl-Meyer Assessment; MAS: Modified Ashworth Scale; MAS-PF: Modified Ashworth Scale – Plantar Flexors; MBI: Modified Barthel Index; MI: Motricity Index; MRC: Medical Research Council Scale; MTS: Modified Tardieu Scale; NRS: Numeric Rating Scale (Pain); PRS: Physician's Rating Scale; RMA: Rivermead Motor Assessment; RMI: Rivermead Mobility Index; ROM: Range of Motion; sEMG: Surface Electromyography; SFS: Spasm Frequency Scale; SIS: Stroke Impact Scale; TUG/TUGT: Timed Up and Go (Test); VAS: Visual Analog Scale; VO₂: Oxygen Consumption.

**Table 2.** Main findings of included studies.

Outcome	Instrument	Reference	Timepoint	*P* value
Spasticity	Modified Ashworth Scale (MAS)	Kaji et al. (2010)	Week 4	<0.001
		Kaji et al. (2010)	Week 8	<0.001
		Kaji et al. (2010)	Week 12	<0.001
		Tok et al. (2012)	Week 8	<0.05
		Esquenazi et al. (2018)	Week 4	<0.05
		Wein et al. (2018)	Week 4–6	0.01
		Kerzoncuf et al. (2020)	Week 4–6	0.035–0.049
		Pittock et al. (2003)	Week 4	0.0002
		Pittock et al. (2003)	Week 8	0.0016
		Pittock et al. (2003)	Week 12	0.0188
	7-point physician scale (Toe)	Wein et al. (2018)	Week 4–6	Not reported
Walk Test	10-Meter Walk Test (10MWT)	Tok et al. (2012)	Week 8	<0.05
		Masakado et al. (2016)	Week 4	≥1
		Masakado et al. (2016)	Week 6	≥1
		Masakado et al. (2016)	Week 8	≥1
		Masakado et al. (2016)	Week 12	≥1
		Tenniglo et al. (2023)	Week 4–6	<0.05
	6-Minute Walk Test (6MWT)	Tenniglo et al. (2023)	Week 4–6	<0.05
		Tok et al. (2012)	Week 8	<0.05
	Gait speed (m/s)	Yu et al. (2023)	Week 12	<0.05
		Tenniglo et al. (2023)	Week 4–6	<0.05
		Tao et al. (2015)	Week 8	<0.05
	Physician Rating Scale (PRS)	Kaji et al. (2010)	Week 4–12	≥1
		Masakado et al. (2016)	Week 4–12	≥1
Sensorimotor impairment	Fugl-Meyer Assessment (FMA)	Tao et al. (2015)	Week 8	<0.05
		Yu et al. (2023)	Week 4	<0.05
		Yu et al. (2023)	Week 12	<0.05
	Lower Extremity Fugl-Meyer Assessment (L-FMA)	Yu et al. (2023)	Week 4	<0.05
		Yu et al. (2023)	Week 12	<0.05
	Motricity Index (MI)	Tenniglo et al. (2023)	Week 4–6	≥1
	Medical Research Council Scale (MRC)	Tenniglo et al. (2023)	Week 4–6	NS
	Rivermead Mobility Index (RMI)	Tenniglo et al. (2023)	Week 4–6	NS
Severity of Illness	CGI – Clinical Global Impression	Kaji et al. (2010)	Week 4	0.016
		Kaji et al. (2010)	Week 8	0.028
		Kaji et al. (2010)	Week 12	0.048
		Esquenazi et al. (2018)	Week 4,6	<0.05
		Wein et al. (2018)	Week 4–6	0.01
Balance	Timed Up and Go Test (TUG)	Yu et al. (2023)	Week 4	<0.05
		Yu et al. (2023)	Week 12	<0.05
		Tao et al. (2015)	Week 8	<0.05
		Tenniglo et al. (2023)	Week 4–6	<0.05
	Force plate (AMTI)	Kerzoncuf et al. (2020)	Week 4–6	0.019
Activities of Daily Living	Modified Barthel Index (MBI)	Tao et al. (2015)	Week 8	<0.05
Goal Attainment	Goal Attainment Scale (GAS)	Esquenazi et al. (2018)	Week 8,12	<0.05
		Wein et al. (2018)	Week 8,12	0.04
Falls	Self-report	Kerzoncuf et al. (2020)	Pre-post	0.006

Statistical analysis was performed using Review Manager (RevMan), version 5.4, utilizing mean differences and standard deviations for continuous variables. Results were expressed as pooled means, effect sizes, and corresponding 95% confidence intervals. To assess heterogeneity among studies, Cochran's Q test and the *I*^2^ index were applied, with values of 25%, 50%, and 75% considered indicative of low, moderate, and high heterogeneity, respectively.^21^ Given the diversity of interventions and study populations analyzed, a random-effects model was applied to provide a more comprehensive and realistic estimate of the combined effects. Observed heterogeneity ranged from low to moderate, and all eligible articles were retained in the meta-analysis.

## Results

The process of identification, screening, and inclusion of articles is summarized in Figure 1, according to the PRISMA 2020 flow diagram, resulting in the final inclusion of 11 studies in the review.

Table 1 presents the main characteristics and data extraction from the studies included in this review, focusing on the effectiveness of BTX in the lower limb after stroke. The table summarizes details on study design, participant demographics, intervention protocols (type and dose of toxin, target muscles), and the main assessment tools used to evaluate the outcomes.^22–32^

### Characteristics of the included studies

Among the 11 studies analyzed on the effectiveness of BTX in the lower limb after stroke, there is a temporal distribution that demonstrates the growing scientific production on this topic over the past two decades. Although the search was conducted without time limits, the earliest publication in the sample dates back to 2003.^24^ There is a higher concentration of studies published between 2015 and 2023, reflecting increased interest and methodological advances in the field.

**Figure 2.**
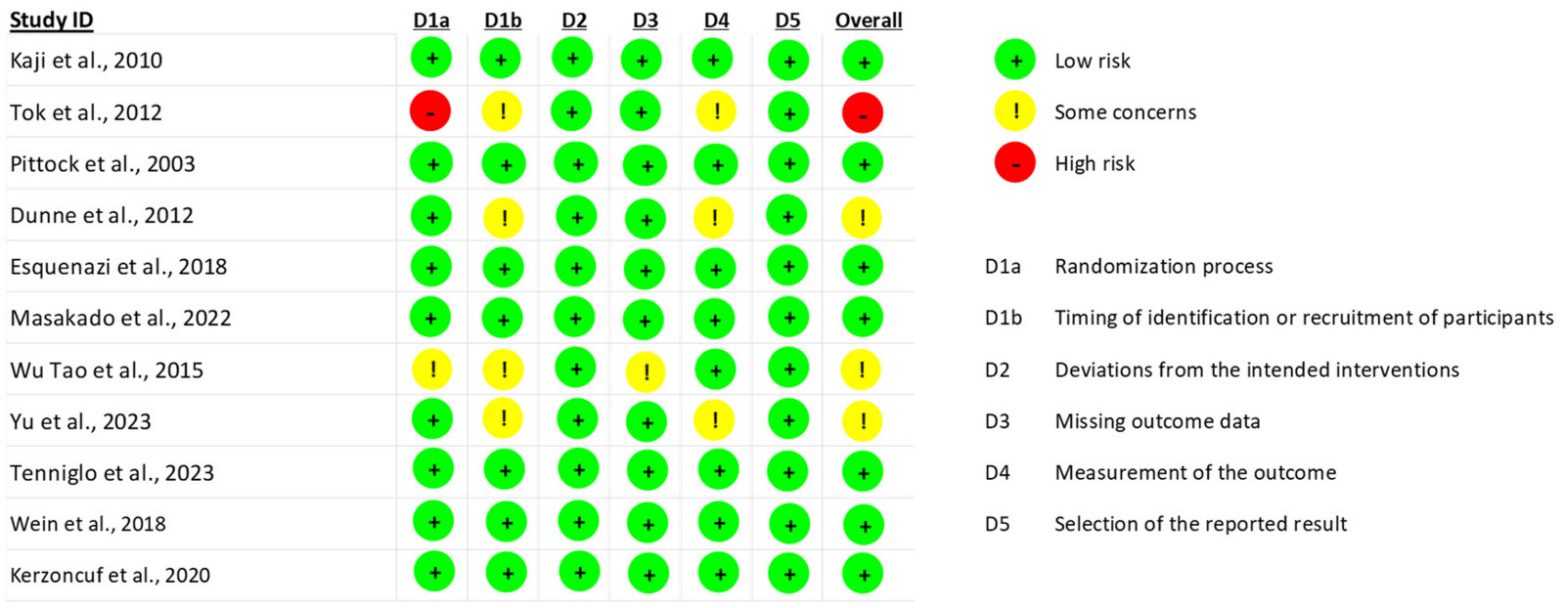
Risk of bias assessment (RoB 2).

Regarding the methodological quality of the included studies (Figure 2), most trials exhibited robust designs, such as randomization, control groups, and double-blind assessment; however, not all fully met the essential criteria to minimize the risk of bias. Some studies^23,25,28,29^ showed limitations in the blinding of participants, healthcare professionals, and/or outcome assessors.

### Characteristics of the participants

The included studies comprised a total of 1740 adult participants, with a mean age ranging from approximately 50–62 years, and a predominance of males (1088). There was significant variability in the number of participants per study, from small trials with about 23–25 patients^23,25,28,29^ to large multicenter studies with more than 200 participants.^24,26,27,31^ In most studies, there was a predominance of ischemic stroke, focusing on patients in the chronic phase, generally with more than 6 months poststroke,^22,27,30–32^ although some studies included participants in the subacute phase (up to 6 months after stroke).^28,29^

The initial degree of spasticity among participants was generally moderate to severe, defined mainly by the Modified Ashworth Scale^33^ (MAS ≥2 or ≥3).^22,24,27,30–32^ The degree of poststroke sequelae was described variably among the included studies. The laterality of impairment was reported in some studies, covering both right and left hemiparesis.^23,25,26,29–32^

### Characteristics of Intervention

The intervention protocols used in the included studies showed some variability regarding the type of BTX administered, dosage, target muscle groups, and method of application. Most trials used onabotulinumtoxinA (BOTOX^®^, Allergan),^22,23,25,26,28–32^ while some studies used incobotulinumtoxinA (Xeomin^®^)^27^ and abobotulinumtoxinA (Dysport^®^).^24^ Total doses ranged from 100 to 400 units, with the majority of studies administering the injections in a single intramuscular session.^22,24,26–28^^,30–32^ In most studies,^22–28^^,30–32^ the control group received placebo (saline solution), administered in an indistinguishable manner from the BTX.

**Figure 3.**
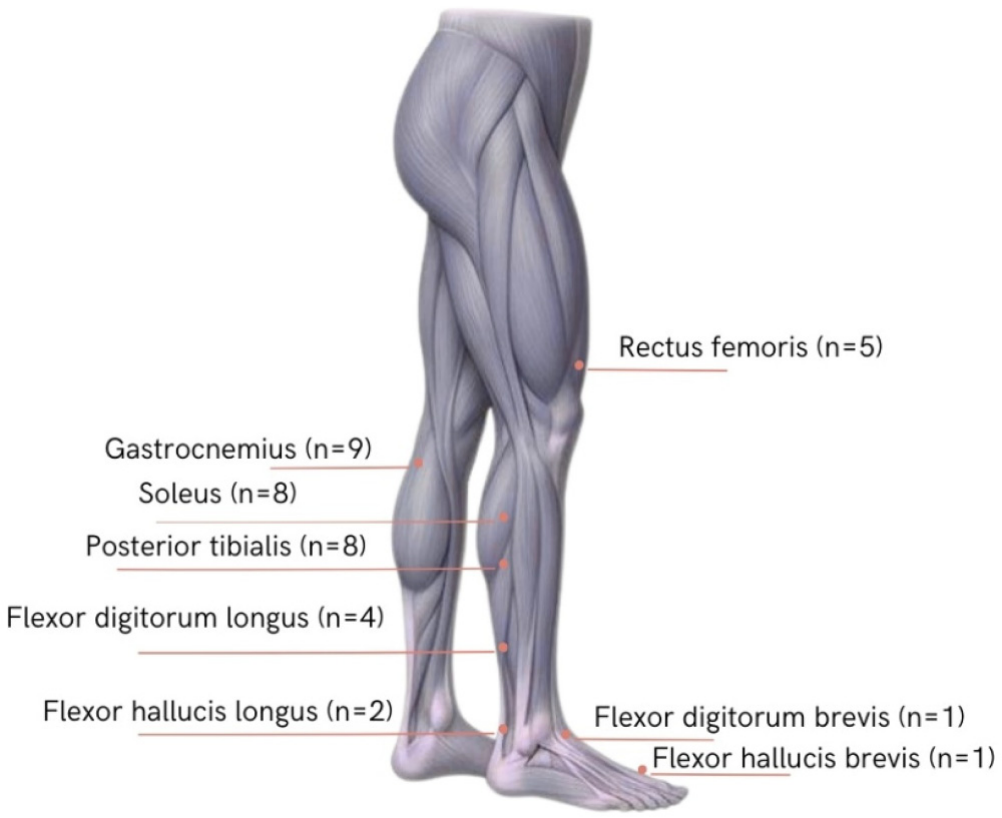
Muscles treated in studies of lower limb Botulinum toxin (Author-generated figure).

The most frequently treated muscles with toxin were the gastrocnemius,^22,24–29^^,31,32^ followed by the soleus^22,24–28^^,31,32^ and posterior tibialis^22,25–29^^,31,32^ (Figure 3). Other muscles were included in a smaller number of studies.

Several instruments were used in the analyzed studies to assess clinical outcomes (Table 2). The MAS was the instrument of choice for evaluating muscle spasticity and was present in almost all of the clinical trials analyzed.^22–32^ For gait assessment, the 10-Meter Walk Test (10MWT) and the 6-Minute Walk Test were applied.^23,26,27,29,30^ Sensorimotor deficits were evaluated using the Fugl-Meyer Assessment (FMA), the Lower Extremity FMA, the Motricity Index, the Medical Research Council Scale, and the Rivermead Mobility Index.^28–30^ Balance was assessed using the Timed Up and Go Test (TUG), which measures dynamic balance and fall risk, and the force plate (AMTI), used for the assessment of postural control.^29,30,32^ The Clinical Global Impression was used to analyze overall clinical progress.^22,26,27,31^ Independence in activities of daily living was measured in only one study using the Modified Barthel Index.^28^

**Figure 4.**
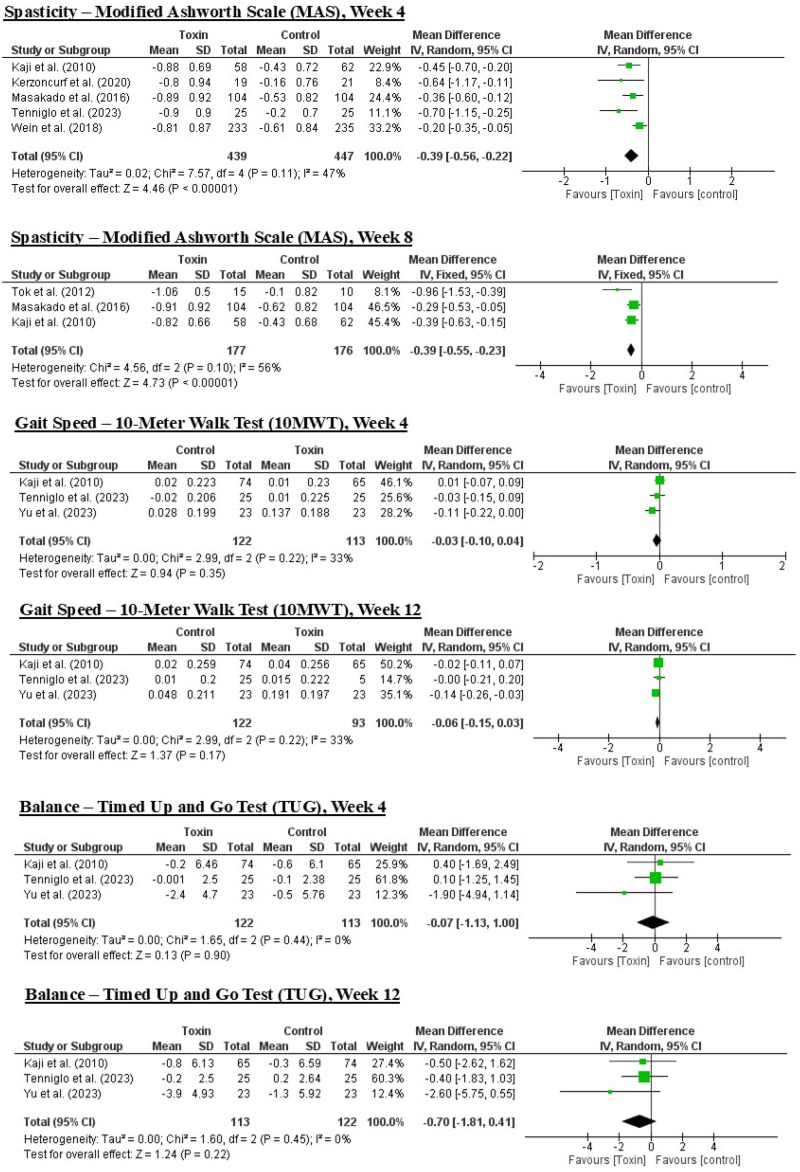
Forest plot of the included studies.

The distribution of effect sizes, confidence intervals, and study heterogeneity are summarized in the forest plots presented in Figure 4. This analysis was performed only for the outcomes for which meta-analysis was possible, namely spasticity MAS, gait speed (10MWT), and balance (TUG).

In the forest plots related to spasticity (MAS),^22,23,26,27,29,30,31^ a statistically significant overall effect favoring the intervention group was observed, with moderate heterogeneity (*I*^2^ = 47% at week 4 and *I*^2^ = 56% at week 8).

In contrast, for the outcomes of gait speed (10MWT) and balance (TUG), the forest plots showed aggregated effect sizes close to zero, with no statistically significant differences between groups. In these cases, heterogeneity was low to moderate (*I*^2^ between 0% and 33%).^23,29,30^

## Discussion

The main objective of this systematic review and meta-analysis was to evaluate the effectiveness of BTX in functional improvement of the lower limb in adults after stroke, considering only clinical trials. This methodological approach clearly distinguishes the present review from previous works, which often included studies with multiple combined interventions, making it difficult to analyze the isolated effect of BTX.^13–17^ Consequently, the number of studies eligible for this review was relatively small, 11 in total, with only 7 included in the meta-analysis. While this methodological choice enhances internal validity, it limits applicability to routine practice, where BTX is rarely delivered in isolation. This may partly account for the limited functional gains identified, as sustained improvements often require repeated injections integrated with rehabilitation.^31^ Moreover, several trials used functional measures that are not specific to lower-limb spasticity, whereas instruments such as the Leg Activity Measure may offer greater sensitivity.^32^

The chronological analysis shows that the included studies were published between 2003 and 2023, with a marked increase in publications from 2015 onward, reflecting growing interest in the use of BTX in poststroke rehabilitation.^22–32^ Variability in the participants of the analyzed studies is a central aspect to consider when validating the results, especially in terms of severity and location of spasticity, type and time since stroke, stroke laterality, sex of the participants, and age range. Previous studies have suggested that the therapeutic response may be influenced by these factors,^8,13,30^ and the lack of subgroup analysis based on these parameters limits the understanding of the intervention's effects.

Although the focus of this review was on lower limb intervention, there was significant variability in the selection of target muscle groups. There was a predominance of interventions targeting distal muscles (gastrocnemius, soleus, tibialis posterior), reflecting their clinical relevance for equinovarus correction and gait pattern control. However, this approach may not address proximal deficits that also limit gait.^22,24–29^^,33,34^

Regarding outcomes, the studies included in this review consistently demonstrate that BTX has a significant effect in reducing lower limb spasticity after stroke.^22,23,26,27,29,30,33^ On the other hand, there is a trend toward improvements in gait^23,26,27,29,30^; however, the gains were not consistent across all studies.^25,27,29,30,34^ Similarly, some results related to sensorimotor function^28,29,30^ and functional balance^29,30,34^ were generally positive.

Although gait and balance differences were nonsignificant, their clinical relevance remains unclear. Future studies should define minimally important differences and report the proportion of responders to improve applicability. Improvements were also observed in domains such as functional independence,^28^ clinical severity assessment,^22,26,27,33^ goal attainment,^26,33^ and reduction in falls,^34^ although these results were limited to a few isolated studies.

The results of the meta-analysis confirmed a statistically significant reduction in lower limb spasticity (MAS) at weeks 4 and 8 after administration of BTX,^22,23,25,27,29,30^ with a favorable effect for the intervention group, despite moderate heterogeneity among studies. On the other hand, no significant differences were found regarding gait^22,28,29^ and balance,^22,28,29^ with effect sizes close to zero and low to moderate heterogeneity. These findings are consistent with the international literature, suggesting that the reduction of spasticity does not always translate into immediate functional gains, and highlighting the importance of addressing other components of poststroke motor impairment.^14–17^

In this context, publications continue to reinforce the role of BTX as the standard treatment for focal spasticity,^1,8,9,12,14,15^ although they recognize that the evidence for sustainable functional improvement remains limited and dependent on complementary therapies. It should be emphasized that spasticity is only one component of poststroke motor impairment,^22,23,33^ as other contributing factors exist, including weakness and deficits in selective motor control, among others.^1,8,9^ Optimization of dose, dilution and injection sites, coupled with goal-oriented rehabilitation (e.g., task-specific gait training and orthosis adjustment), may help translate tone reduction into functional gains.^1,9,11,12^

Future trials should use more homogeneous, rigorous designs with representative samples and longer follow-up. Subgroup analyses are essential to identify responders and to understand which factors help convert tone reduction into functional improvement.

Among the main limitations of this review, the first is the small number of eligible studies, reflecting the scarcity of RCTs that evaluate the isolated use of BTX in the lower limb after stroke. Secondly, heterogeneity among studies regarding participants, intervention protocols, and assessment instruments may have led to some bias in the results. Thirdly, there is the potential for language bias in the included publications. Given the limited number of studies per outcome in the meta-analysis, formal statistical tests for publication bias were not conducted owing to insufficient power. Finally, most participants in the included studies were in the chronic poststroke phase and had moderate-to-severe spasticity, which limits the generalizability of the findings to earlier poststroke phases or to populations with other types of impairments.

## Supplemental Material

sj-docx-2-cre-10.1177_02692155261417499 - Supplemental material for Efficacy of botulinum toxin for poststroke lower limb: a systematic review and meta-analysis Supplemental material, sj-docx-2-cre-10.1177_02692155261417499 for Efficacy of botulinum toxin for poststroke lower limb: a systematic review and meta-analysis by Carla Sílvia Fernandes, Andreia Maria Novo Lima, Maria Teresa Moreira, Salome Sobral Sousa and Maria Salomé Ferreira in Clinical Rehabilitation

sj-docx-3-cre-10.1177_02692155261417499 - Supplemental material for Efficacy of botulinum toxin for poststroke lower limb: a systematic review and meta-analysis Supplemental material, sj-docx-3-cre-10.1177_02692155261417499 for Efficacy of botulinum toxin for poststroke lower limb: a systematic review and meta-analysis by Carla Sílvia Fernandes, Andreia Maria Novo Lima, Maria Teresa Moreira, Salome Sobral Sousa and Maria Salomé Ferreira in Clinical Rehabilitation

sj-docx-4-cre-10.1177_02692155261417499 - Supplemental material for Efficacy of botulinum toxin for poststroke lower limb: a systematic review and meta-analysis Supplemental material, sj-docx-4-cre-10.1177_02692155261417499 for Efficacy of botulinum toxin for poststroke lower limb: a systematic review and meta-analysis by Carla Sílvia Fernandes, Andreia Maria Novo Lima, Maria Teresa Moreira, Salome Sobral Sousa and Maria Salomé Ferreira in Clinical Rehabilitation
