# Supplementary material for: Efficacy of botulinum toxin for poststroke lower limb: a systematic review and meta-analysis
Source: Clin Rehabil. 2026 Feb 13;40(7):893–905. doi: 10.1177/02692155261417499 (PMC13283505; doi:10.1177/02692155261417499)
Supplement: sj-docx-2-cre-10.1177_02692155261417499 - Supplemental material for Efficacy of botulinum toxin for poststroke lower limb: a systematic review and meta-analysis [file sj-docx-2-cre-10.1177_02692155261417499.docx]

**supplementary material**

| **MEDLINE®** |
| --- |
| (((MM "Cerebrovascular Disorders") OR (MM "Cerebral Hemorrhage") OR (MM "Brain Ischemia") OR (MH "Stroke") OR (MM "Brain Infarction") OR (MM "Cerebral Infarction") OR (MM "Subarachnoid Hemorrhage") OR (MM "Brain Injuries") OR (MH "Brain Damage, Chronic") OR (MM "Carotid Artery Diseases") OR (MH "Cerebral Arterial Diseases+") OR (MM "Intracranial Arterial Diseases") OR (MM "Intracranial Arteriovenous Malformations") OR (MH "Intracranial Embolism and Thrombosis+") OR (MM "Intracranial Hemorrhages") OR (MM "Ischemic Attack, Transient") OR (“cerebral haemorrhage”) OR (“cerebrovascular accident*”) OR (“cerebral vascular event*”) OR (“poststroke”) OR (“post-stroke”) OR (“after stroke”)) AND ((MM "Botulinum Toxins") OR (MM "Botulinum Toxins, Type A") OR ("botulinum toxin") OR ("botulinum toxin type A") OR ("botox") OR ("onabotulinumtoxinA") OR ("botulinum neurotoxin") OR ("clostridium botulinum toxin")) AND ((MM "Lower Extremity") OR (MM "Leg") OR (MM "Foot") OR ("lower extremity") OR ("lower extremities") OR ("lower limb") OR ("lower limbs") OR ("leg") OR ("legs") OR ("foot") OR ("feet") OR ("inferior limb") OR ("inferior limbs") OR (MM "Knee") OR ("knee") OR ("knees") OR ("quadriceps") OR ("gastrocnemius") OR ("soleus") OR ("hamstring") OR ("hamstrings")) AND ((MH "Randomized Controlled Trials as Topic") OR ("Randomized Controlled Trial") OR ("randomized")OR ("randomised") OR ("randomly") OR ("RCT") OR ("controlled clinical trial") OR ("control group"))) |
|  |
| **CINAHL®** |
| (((MM "Subarachnoid Hemorrhage") OR (MM "Brain Damage, Chronic") OR (MM "Brain Injuries") OR (MH "Stroke+") OR (MM "Carotid Artery Diseases") OR (MM "Intracranial Arterial Diseases") OR (MM "Cerebral Arterial Diseases") OR (MM "Intracranial Embolism and Thrombosis") OR (MM "Intracranial Hemorrhage") OR (MM "Cerebral Ischemia, Transient") OR (“brain ischemia”) OR (“cerebrovascular accident*”) OR (“cerebral vascular event*”) OR (“poststroke”) OR (“post-stroke”) OR (“after stroke”) OR ("brain infarction”) OR (“intracranial arteriovenous malformation*”) OR (“cerebral haemorrhage”)) AND ((MM "Botulinum Toxins") OR (MM "Botulinum Toxins, Type A") OR ("botulinum toxin") OR ("botulinum toxin type A") OR ("botox") OR ("onabotulinumtoxinA") OR ("botulinum neurotoxin") OR ("clostridium botulinum toxin")) AND ((MM "Lower Extremity") OR (MM "Leg") OR (MM "Foot") OR ("lower extremity") OR ("lower extremities") OR ("lower limb") OR ("lower limbs") OR ("leg") OR ("legs") OR ("foot") OR ("feet") OR ("inferior limb") OR ("inferior limbs") OR (MM "Knee") OR ("knee") OR ("knees") OR ("quadriceps") OR ("gastrocnemius") OR ("soleus") OR ("hamstring") OR ("hamstrings")) AND ((MH "Randomized Controlled Trials as Topic") OR ("Randomized Controlled Trial") OR ("randomized") OR ("randomised") OR ("randomly") OR ("RCT") OR ("controlled clinical trial") OR ("control group"))) |
|  |
| **Psychology and Behavioral Sciences Collection** |
| (((DE "CEREBRAL hemorrhage") OR (DE "CEREBRAL ischemia") OR (DE "TRANSIENT ischemic attack") OR (DE "CEREBROVASCULAR disease") OR (DE "SUBARACHNOID hemorrhage") OR (DE "BRAIN damage") OR (DE "BRAIN injuries") OR (DE "ALTERNATIVE treatment for stroke") OR (DE "STROKE patients -- Rehabilitation") OR (DE "CEREBRAL infarction") OR (DE "CAROTID artery diseases") OR (DE "INTRACRANIAL arterial diseases") OR (DE "CEREBRAL arterial diseases") OR (DE "CEREBRAL arteriovenous malformations") OR (DE "BRAIN blood-vessel abnormalities") OR (“cerebrovascular disorder*”) OR (“cerebral haemorrhage”) OR (“brain ischemia”) OR (“cerebrovascular accident*”) OR (“cerebral vascular event*”) OR (“stroke”) OR (“poststroke”) OR (“post-stroke") OR (“after stroke”) OR (“brain infarction”) OR (“intracranial arteriovenous malformations”) OR (“intracranial embolism and thrombosis") OR ("intracranial hemorrhages")) AND ((DE "Botulinum Toxins") OR (DE "Botulinum Toxins, Type A") OR ("botulinum toxin") OR ("botulinum toxin type A") OR ("botox") OR ("onabotulinumtoxinA") OR ("botulinum neurotoxin") OR ("clostridium botulinum toxin")) AND ((DE "Lower Extremity") OR (DE "Leg") OR (DE "Foot") OR ("lower extremity") OR ("lower extremities") OR ("lower limb") OR ("lower limbs") OR ("leg") OR ("legs") OR ("foot") OR ("feet") OR ("inferior limb") OR ("inferior limbs") OR (DE "Knee") OR ("knee") OR ("knees") OR ("quadriceps") OR ("gastrocnemius") OR ("soleus") OR ("hamstring") OR ("hamstrings")) AND (("randomized controlled trial") OR ("randomised controlled trial") OR ("randomized") OR ("randomised") OR ("randomly") OR ("RCT") OR ("controlled clinical trial") OR ("control group"))) |
|  |
| **SPORTS** |
| (((DE "CEREBROVASCULAR disease") OR (DE "CEREBRAL hemorrhage") OR (DE "BRAIN damage") OR (DE "BRAIN injuries") OR (DE "STROKE") OR (DE "CEREBROVASCULAR disease") OR (“cerebrovascular disorder*”) OR (“cerebral haemorrhage”) OR (“cerebral ischemia”) OR (“brain ischemia”) OR (“cerebrovascular accident*”) OR (“cerebral vascular event*”) OR (“subarachnoid haemorrhage”) OR (“poststroke”) OR ("post-stroke") OR (“after stroke”) OR (“brain infarction”) OR ("carotid artery diseases") OR ("intracranial arterial diseases") OR ("cerebral arterial diseases") OR (“intracranial arteriovenous malformation*”) OR ("intracranial embolism and thrombosis") OR ("intracranial hemorrhage*")) AND ((DE "Botulinum Toxins") OR (DE "Botulinum Toxins, Type A") OR ("botulinum toxin") OR ("botulinum toxin type A") OR ("botox") OR ("onabotulinumtoxinA") OR ("botulinum neurotoxin") OR ("clostridium botulinum toxin")) AND ((DE "Lower Extremity") OR (DE "Leg") OR (DE "Foot") OR ("lower extremity") OR ("lower extremities") OR ("lower limb") OR ("lower limbs") OR ("leg") OR ("legs") OR ("foot") OR ("feet") OR ("inferior limb") OR ("inferior limbs") OR (DE "Knee") OR ("knee") OR ("knees") OR ("quadriceps") OR ("gastrocnemius") OR ("soleus") OR ("hamstring") OR ("hamstrings")) AND (("randomized controlled trial") OR ("randomised controlled trial") OR ("randomized") OR ("randomised") OR ("randomly") OR ("RCT") OR ("controlled clinical trial") OR ("control group"))) |
|  |
| **SCOPUS** |
| (((TITLE-ABS-KEY("cerebrovascular disorder*") OR TITLE-ABS-KEY("cerebral haemorrhage") OR TITLE-ABS-KEY("cerebral hemorrhage") OR TITLE-ABS-KEY("cerebral ischemia") OR TITLE-ABS-KEY("brain ischemia") OR TITLE-ABS-KEY("cerebrovascular accident*") OR TITLE-ABS-KEY("cerebral vascular event*") OR TITLE-ABS-KEY("subarachnoid haemorrhage") OR TITLE-ABS-KEY("brain damage") OR TITLE-ABS-KEY("brain injurie*") OR TITLE-ABS-KEY("stroke") OR TITLE-ABS-KEY("poststroke") OR TITLE-ABS-KEY("post-stroke") OR TITLE-ABS-KEY("after stroke") OR TITLE-ABS-KEY("brain infarction") OR TITLE-ABS-KEY("carotid artery disease*") OR TITLE-ABS-KEY("intracranial arterial disease*") OR TITLE-ABS-KEY("cerebral arterial disease*") OR TITLE-ABS-KEY("intracranial arteriovenous malformation*") OR TITLE-ABS-KEY("intracranial embolism and thrombosis") OR TITLE-ABS-KEY("intracranial haemorrhage*")) AND ((TITLE-ABS-KEY("botulinum toxin") OR TITLE-ABS-KEY("botulinum toxins") OR TITLE-ABS-KEY("botulinum toxin type A") OR TITLE-ABS-KEY("clostridium botulinum A toxin") OR TITLE-ABS-KEY("botulinum neurotoxin A") OR TITLE-ABS-KEY("botulinum A toxin") OR TITLE-ABS-KEY("botox") OR TITLE-ABS-KEY("onabotulinumtoxinA") OR TITLE-ABS-KEY("botulinum neurotoxin") OR TITLE-ABS-KEY("clostridium botulinum toxin")) AND (TITLE-ABS-KEY("lower extremity") OR TITLE-ABS-KEY("lower extremities") OR TITLE-ABS-KEY("lower limb") OR TITLE-ABS-KEY("lower limbs") OR TITLE-ABS-KEY("leg") OR TITLE-ABS-KEY("legs") OR TITLE-ABS-KEY("foot") OR TITLE-ABS-KEY("feet") OR TITLE-ABS-KEY("inferior limb") OR TITLE-ABS-KEY("inferior limbs") OR TITLE-ABS-KEY("knee") OR TITLE-ABS-KEY("knees") OR TITLE-ABS-KEY("quadriceps") OR TITLE-ABS-KEY("gastrocnemius") OR TITLE-ABS-KEY("soleus") OR TITLE-ABS-KEY("hamstring") OR TITLE-ABS-KEY("hamstrings")) AND (TITLE-ABS-KEY("randomized controlled trial") OR TITLE-ABS-KEY("randomised controlled trial") OR TITLE-ABS-KEY("randomized") OR TITLE-ABS-KEY("randomised") OR TITLE-ABS-KEY("randomly") OR TITLE-ABS-KEY("RCT") OR TITLE-ABS-KEY("controlled clinical trial") OR TITLE-ABS-KEY("control group"))) |
| **Cochrane** |
| (((TITLE-ABS-KEY("cerebrovascular disorder*") OR TITLE-ABS-KEY("cerebral haemorrhage") OR TITLE-ABS-KEY("cerebral hemorrhage") OR TITLE-ABS-KEY("cerebral ischemia") OR TITLE-ABS-KEY("brain ischemia") OR TITLE-ABS-KEY("cerebrovascular accident*") OR TITLE-ABS-KEY("cerebral vascular event*") OR TITLE-ABS-KEY("subarachnoid haemorrhage") OR TITLE-ABS-KEY("brain damage") OR TITLE-ABS-KEY("brain injurie*") OR TITLE-ABS-KEY("stroke") OR TITLE-ABS-KEY("poststroke") OR TITLE-ABS-KEY("post-stroke") OR TITLE-ABS-KEY("after stroke") OR TITLE-ABS-KEY("brain infarction") OR TITLE-ABS-KEY("carotid artery disease*") OR TITLE-ABS-KEY("intracranial arterial disease*") OR TITLE-ABS-KEY("cerebral arterial disease*") OR TITLE-ABS-KEY("intracranial arteriovenous malformation*") OR TITLE-ABS-KEY("intracranial embolism and thrombosis") OR TITLE-ABS-KEY("intracranial haemorrhage*")) AND ((TITLE-ABS-KEY("botulinum toxin") OR TITLE-ABS-KEY("botulinum toxins") OR TITLE-ABS-KEY("botulinum toxin type A") OR TITLE-ABS-KEY("clostridium botulinum A toxin") OR TITLE-ABS-KEY("botulinum neurotoxin A") OR TITLE-ABS-KEY("botulinum A toxin") OR TITLE-ABS-KEY("botox") OR TITLE-ABS-KEY("onabotulinumtoxinA") OR TITLE-ABS-KEY("botulinum neurotoxin") OR TITLE-ABS-KEY("clostridium botulinum toxin")) AND (TITLE-ABS-KEY("lower extremity") OR TITLE-ABS-KEY("lower extremities") OR TITLE-ABS-KEY("lower limb") OR TITLE-ABS-KEY("lower limbs") OR TITLE-ABS-KEY("leg") OR TITLE-ABS-KEY("legs") OR TITLE-ABS-KEY("foot") OR TITLE-ABS-KEY("feet") OR TITLE-ABS-KEY("inferior limb") OR TITLE-ABS-KEY("inferior limbs") OR TITLE-ABS-KEY("knee") OR TITLE-ABS-KEY("knees") OR TITLE-ABS-KEY("quadriceps") OR TITLE-ABS-KEY("gastrocnemius") OR TITLE-ABS-KEY("soleus") OR TITLE-ABS-KEY("hamstring") OR TITLE-ABS-KEY("hamstrings")) AND (TITLE-ABS-KEY("randomized controlled trial") OR TITLE-ABS-KEY("randomised controlled trial") OR TITLE-ABS-KEY("randomized") OR TITLE-ABS-KEY("randomised") OR TITLE-ABS-KEY("randomly") OR TITLE-ABS-KEY("RCT") OR TITLE-ABS-KEY("controlled clinical trial") OR TITLE-ABS-KEY("control group"))) |
