# Supplementary material for: Efficacy of botulinum toxin for poststroke lower limb: a systematic review and meta-analysis
Source: Clin Rehabil. 2026 Feb 13;40(7):893–905. doi: 10.1177/02692155261417499 (PMC13283505; doi:10.1177/02692155261417499)
Supplement: sj-docx-3-cre-10.1177_02692155261417499 - Supplemental material for Efficacy of botulinum toxin for poststroke lower limb: a systematic review and meta-analysis [file sj-docx-3-cre-10.1177_02692155261417499.docx]

**Table 1.** Data Extraction

| **Author**  **(Year)**  **Country** | **Study Design** | **Participants** | | | | | | | **Intervention** | | | **Control Group** | **Assessment Tools** |
| --- | --- | --- | --- | --- | --- | --- | --- | --- | --- | --- | --- | --- | --- |
|  |  | N | Mean age (±SD) | Sex | Stroke phase | Stroke type | Affected side | Degree of spasticity | Type of toxin | Dose | Muscle group(s) |  |  |
| Kaji et al. (2010) [22]  Japan | Multicenter, randomized, double-blind, placebo-controlled trial | 120 (Toxin: 58; Placebo: 62) | 62.4 ± 8.7 (Toxin); 62.5 ± 9.3 (Placebo) | 8F/50M (Toxin); 16F/46M (Placebo) | Chronic (≥6 months; mean 80.8 ± 72.8 months for Toxin; 72.0 ± 60.3 months for placebo) | Not specified | Not specified | MAS ~3.8(Toxin), 3.2 (Placebo) | OnabotulinumtoxinA (BOTOX®, Allergan) | 300 U total  75 U each muscle, single session | Medial gastrocnemius, lateral gastrocnemius, soleus, tibialis posterior; | Placebo (saline, identical injection) | MAS, PRS, GS, CGI |
| Tok et al. (2012) [23]  Turkey | Randomized, double-blind, placebo-controlled trial | N = 25 (toxin A: 15; Placebo: 10) | 53.9 ± 14.7 (Toxin), 59.0 ± 8.1 (Placebo) | Toxin  7F/8M, Placebo 4F/6M | Chronic ≥6 months; duration Toxin 14.6 ± 7.2 Placebo 13.4 ± 7.0 | Ischemic 80%, Hemorrhagic 20% | Toxin 9R/6L, Placebo 5R/5L | (rectus femoris) 2.46 ± 0.63 (Toxin), 2.40 ± 0.51 (Placebo) | Onabotulinum toxin A (BOTOX®, Allergan), | mean dose 113.3 ± 12.9 U (range 100–125 U), single injection | Rectus femoris | Placebo (saline, identical injection) | MAS, 10MWT, 6MWT, 3D Gait, VO₂ |
| Pittock et al.  (2003) [24]  Multicenter | Randomized, double-blind, placebo-controlled trial | N = 234 Placebo: 55  Dysport 500U: 59  Dysport 1000U: 60  Dysport 1500U: 60 | Placebo: 55.9 (±11.4)  500U: 56.4 (±12.8)  1000U: 54.8 (±13.6)  1500U: 54.7 (±10.2) | Placebo: 37/18  500U: 36/23  1000U: 39/21  1500U: 35/25 | Chronic (≥3 months post-stroke) | Intracerebral hemorrhage, subarachnoid hemorrhage, cerebral thrombosis, cerebral embolism | Both sides included | MAS (mean ~3.0, some up to 5) | BoNT-A (Dysport®) | 500, 1000, 1500  Single injection per group | Gastrocnemius, soleus | Placebo (saline, identical injection) | MAS, 2MWT, RMA |
| Dunne et al. (2012) [25]  Australia | Randomized, double-blind, placebo-controlled trial | N = 83  OnabotulinumtoxinA: 54  Placebo: 29 | OnabotulinumtoxinA 57.9 ± 13.8  Placebo 59.5 ± 10.6 | OnabotulinumtoxinA: 76% M  Placebo: 76% M | Chronic Median 2.1 years (mean 3.4 ± 3.8 years), 22.4% < 6 months | 74% ischemic, 26% hemorrhagic (overall) | Right: 60%, Left: 40% | Moderate-severe (Ashworth ≥2) | OnabotulinumtoxinA (Botox) | 200 U or 300 U (pooled in analysis); max 300 U | Tibialis posterior, soleus, medial gastrocnemius, flexor digitorum longus | Placebo (saline, identical injection) | MAS, SFS, PRS, VAS |
| Esquenazi et al.  (2018) [26]  Multicenter | Multicenter, randomized, double-blind, placebo-controlled trial | N = 468 (OnabotulinumtoxinA: 233; Placebo: 235) | 56.0 (±12.6) (ona), 57.0 (±11.9) (placebo) | 148/85 (ona), 155/80 (placebo) | Chronic ≥6 months post-stroke | Not specified | Both | MAS ≥3 (92% baseline MAS=3) | OnabotulinumtoxinA (BOTOX®) | 300–400 (mean 347.5) Single injection | Medial gastrocnemius, lateral gastrocnemius, soleus, tibialis posterior | Placebo (saline, identical injection) | MAS, CGI, GAS, 10MWT |
| Masakado et al.  (2016) [27]  Japan | Randomized, double-blind, placebo-controlled trial | N = 208 IncobotulinumtoxinA: 104 Placebo: 104 | IncobotulinumtoxinA: 59.5 (11.2) Placebo: 58.8 (11.0) | IncobotulinumtoxinA: 74/30 Placebo: 84/20 | Chronic (>6 months) | Ischemic: ~30% Hemorrhagic: ~70% | Unilateral (hemiparesis) | MAS-PF = 3 (severe) | IncobotulinumtoxinA (Xeomin®) | 400 (fixed) | - Medial gastrocnemius  - Lateral gastrocnemius - Soleus  - Tibialis posterior  - Flexor digitorum longus  - Flexor hallucis longus | Placebo (saline, identical injection) | MAS, MAS-PF, 10MWT, PRS, CGI, VAS, NRS |
| Tao et al. (2015) [28]  China | Randomized, double-blind, placebo-controlled trial | N=23  (Toxin: 11; Placebo: 13) | Treatment: 55±12  Control: 58±14 | Treatment: 7/4  Control: 8/4 | Subacute (within 6 weeks) | Ischemic/hemorrhagic (Treatment: 6/5; Control: 7/5) | Not specified | MAS 1–1+ or ankle clonus (+) | Botulinum toxin A (Allergan, Botox) | 200 U | Triceps surae (150 U), posterior tibial (50 U) | Placebo (saline, identical injection) | MAS, FMA, sEMG, MBI |
| Yu et al. (2023) [29]  China | Randomized, double-blind, placebo-controlled trial | N = 46 (Toxin: 23; Placebo: 23) | Control: 54.4 ± 9.8  Experimental: 56.5 ± 6.8 | Control: 8 F / 14 M  Experimental: 5 F / 16 M | Subacute (≤6 months post-stroke) | Cerebral infarct (32), cerebral hemorrhage (14) | Control: 12 L / 10 R  Experimental: 13 L | Slight spasticity at rest: MAS 1–1+ or ankle clonus (+) | ~150–300 IU/muscle (see below) | 200–300 U total | Quadriceps femoris, gastrocnemius, tibialis posterior, flexor hallucis longus, flexor digitorum longus, flexor digitorum brevis, flexor hallucis brevis | Routine rehabilitation | MAS, L-FMA, 10MWT, TUGT |
| Tenniglo et al. (2023) [30  Netherlands | Randomized, double-blind, placebo-controlled trial | N=25 (crossover: BoNT-A and placebo) | 57.4 (12.7) | 19 M / 6 F | Chronic (≥6 months post-stroke) | 17 ischemic / 8 hemorrhagic | 15 left / 10 right | Moderate to severe | OnabotulinumtoxinA (Botox, Allergan) | 200 U (6 x 33U into rectus femoris) | Rectus femoris | Placebo (saline, identical injection) | 3D Gait, 6MWT, 10MWT, TUG, MI, RMI, MRC, VAS, SIS |
| Wein et al. (2018) [31]  Multicente | Randomized, double-blind, placebo-controlled trial | N=468 (233 intervention / 235 placebo) | Intervention: 56.0 (12.6) Placebo: 57.0 (11.9) | Intervention: 148 M / 85 F Placebo: 155 M / 80 F | Chronic (≥3 months after stroke) Mean time since stroke: 67.1 mo (INT), 61.6 mo (PLA) | Not specified | Right only, left only, right arm/leg, left arm/leg | MAS ≥3 | OnabotulinumtoxinA (Botox®) | 300 U | gastrocnemius medial/lateral, soleus, tibialis posterior) + optional muscles, flexors/extensors, rectus femoris) | Placebo (saline, identical injection) | MAS, CGI, GAS, VAS, MTS |
| Kerzoncuf et al.  (2020) [32]  Farnce | Randomized, double-blind, placebo-controlled trial | N=  40 (19 BoNT-A / 21 placebo, analyzed) | BoNT-A: 53.4 ± 14.8  Placebo: 50.7 ± 12.9 | Not specified | Chronic (≥12 months post-stroke, mean ~4–6 yrs) | Ischemic & hemorrhagic (proportion not specified) | Both (left/right, similar) | MAS ≥2 | OnabotulinumtoxinA (Botox®) | Mean 227 U (range 50–300 U) | Soleus (88%), gastrocnemius (73%), tibialis posterior, flexor digitorum longus, flexor/extensor hallucis | Placebo (saline, identical injection) | MAS, ROM, FAC, FIM |
| **Legend:**  3D Gait = Three-Dimensional Gait Analysis; 6MWT = 6-Minute Walk Test; 10MWT = 10-Meter Walk Test; 2MWT = 2-Minute Walk Test; CGI = Clinical Global Impression; FAC = Functional Ambulation Category; FIM = Functional Independence Measure; FMA = Fugl-Meyer Assessment; GAS = Goal Attainment Scale; GS = Gait Speed; L-FMA = Lower-limb Fugl-Meyer Assessment; MAS = Modified Ashworth Scale; MAS-PF = Modified Ashworth Scale – Plantar Flexors; MBI = Modified Barthel Index; MI = Motricity Index; MRC = Medical Research Council Scale; MTS = Modified Tardieu Scale; NRS = Numeric Rating Scale (Pain); PRS = Physician’s Rating Scale; RMA = Rivermead Motor Assessment; RMI = Rivermead Mobility Index; ROM = Range of Motion; sEMG = Surface Electromyography; SFS = Spasm Frequency Scale; SIS = Stroke Impact Scale; TUG/TUGT = Timed Up and Go (Test); VAS = Visual Analogue Scale; VO₂ = Oxygen Consumption. | | | | | | | | | | | | | |
