# Supplementary material for: Efficacy of botulinum toxin for poststroke lower limb: a systematic review and meta-analysis
Source: Clin Rehabil. 2026 Feb 13;40(7):893–905. doi: 10.1177/02692155261417499 (PMC13283505; doi:10.1177/02692155261417499)
Supplement: sj-docx-4-cre-10.1177_02692155261417499 - Supplemental material for Efficacy of botulinum toxin for poststroke lower limb: a systematic review and meta-analysis [file sj-docx-4-cre-10.1177_02692155261417499.docx]

**Table 2**: Main Findings of Included Studies

| **Outcome** | **Instrument** | **Reference** | **Timepoint** | **PValue** |
| --- | --- | --- | --- | --- |
| Spasticity | Modified Ashworth Scale (MAS) | Kaji et al. (2010) | Week 4 | <0.001 |
|  |  | Kaji et al. (2010) | Week 8 | <0.001 |
|  |  | Kaji et al. (2010) | Week 12 | <0.001 |
|  |  | Tok et al. (2012) | Week 8 | <0.05 |
|  |  | Esquenazi et al. (2018) | Week 4 | <0.05 |
|  |  | Wein et al. (2018) | Week 4-6 | 0.01 |
|  |  | Kerzoncuf et al. (2020) | Week 4-6 | 0.035–0.049 |
|  |  | Pittock et al. (2003) | Week 4 | 0.0002 |
|  |  | Pittock et al. (2003) | Week 8 | 0.0016 |
|  |  | Pittock et al. (2003) | Week 12 | 0.0188 |
|  | 7-point physician scale (Toe) | Wein et al. (2018) | Week 4-6 | Not reported |
| Walk Test | 10-Meter Walk Test (10MWT) | Tok et al. (2012) | Week 8 | <0.05 |
|  |  | Masakado et al. (2016) | Week 4 | ≥1 |
|  |  | Masakado et al. (2016) | Week 6 | ≥1 |
|  |  | Masakado et al. (2016) | Week 8 | ≥1 |
|  |  | Masakado et al. (2016) | Week 12 | ≥1 |
|  |  | Tenniglo et al. (2023) | Week 4-6 | <0.05 |
|  | 6-Minute Walk Test (6MWT) | Tenniglo et al. (2023) | Week 4-6 | <0.05 |
|  |  | Tok et al. (2012) | Week 8 | <0.05 |
|  | Gait speed (m/s) | Yu et al. (2023) | Week 12 | <0.05 |
|  |  | Tenniglo et al. (2023) | Week 4-6 | <0.05 |
|  |  | Tao et al. (2015) | Week 8 | <0.05 |
|  | Physician Rating Scale (PRS) | Kaji et al. (2010) | Week 4-12 | ≥1 |
|  |  | Masakado et al. (2016) | Week 4-12 | ≥1 |
| Sensorimotor impairment | Fugl-Meyer Assessment (FMA) | Tao et al. (2015) | Week 8 | <0.05 |
|  |  | Yu et al. (2023) | Week 4 | <0.05 |
|  |  | Yu et al. (2023) | Week 12 | <0.05 |
|  | Lower Extremity Fugl-Meyer Assessment (L-FMA) | Yu et al. (2023) | Week 4 | <0.05 |
|  |  | Yu et al. (2023) | Week 12 | <0.05 |
|  | Motricity Index (MI) | Tenniglo et al. (2023) | Week 4-6 | ≥1 |
|  | Medical Research Council Scale (MRC) | Tenniglo et al. (2023) | Week 4-6 | NS |
|  | Rivermead Mobility Index (RMI) | Tenniglo et al. (2023) | Week 4-6 | NS |
| Severity of Illness | CGI – Clinical Global Impression | Kaji et al. (2010) | Week 4 | 0.016 |
|  |  | Kaji et al. (2010) | Week 8 | 0.028 |
|  |  | Kaji et al. (2010) | Week 12 | 0.048 |
|  |  | Esquenazi et al. (2018) | Week 4,6 | <0.05 |
|  |  | Wein et al. (2018) | Week 4-6 | 0.01 |
| Balance | Timed Up and Go Test (TUG) | Yu et al. (2023) | Week 4 | <0.05 |
|  |  | Yu et al. (2023) | Week 12 | <0.05 |
|  |  | Tao et al. (2015) | Week 8 | <0.05 |
|  |  | Tenniglo et al. (2023) | Week 4-6 | <0.05 |
|  | Force plate (AMTI) | Kerzoncuf et al. (2020) | Week 4-6 | 0.019 |
| Activities of Daily Living | Modified Barthel Index (MBI) | Tao et al. (2015) | Week 8 | <0.05 |
| Goal Attainment | Goal Attainment Scale (GAS) | Esquenazi et al. (2018) | Week 8,12 | <0.05 |
|  |  | Wein et al. (2018) | Week 8,12 | 0.04 |
| Falls | Self-report | Kerzoncuf et al. (2020) | Pre-post | 0.006 |
